# Supplementary figures and images for: Pan-Genomic and Polymorphic Driven Prediction of Antibiotic Resistance in Elizabethkingia
Source: Front Microbiol. 2019 Jul 4;10:1446. doi: 10.3389/fmicb.2019.01446 (PMC6622151; doi:10.3389/fmicb.2019.01446)

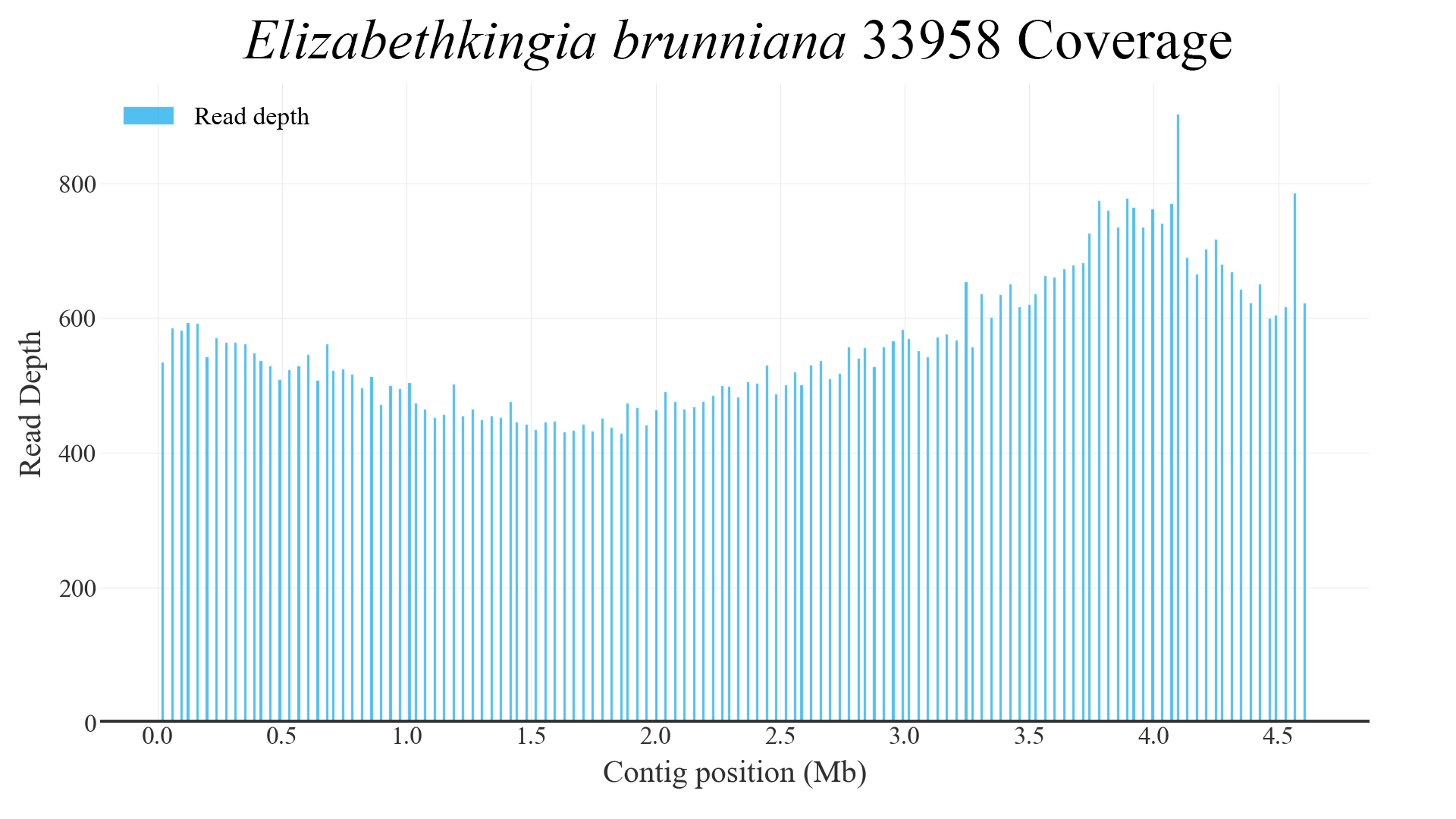

Supplement: FIGURE S1 — The Nanopore R9.4 read coverage for E. bruuniana ATCC 33958. [file Image_1.tif]

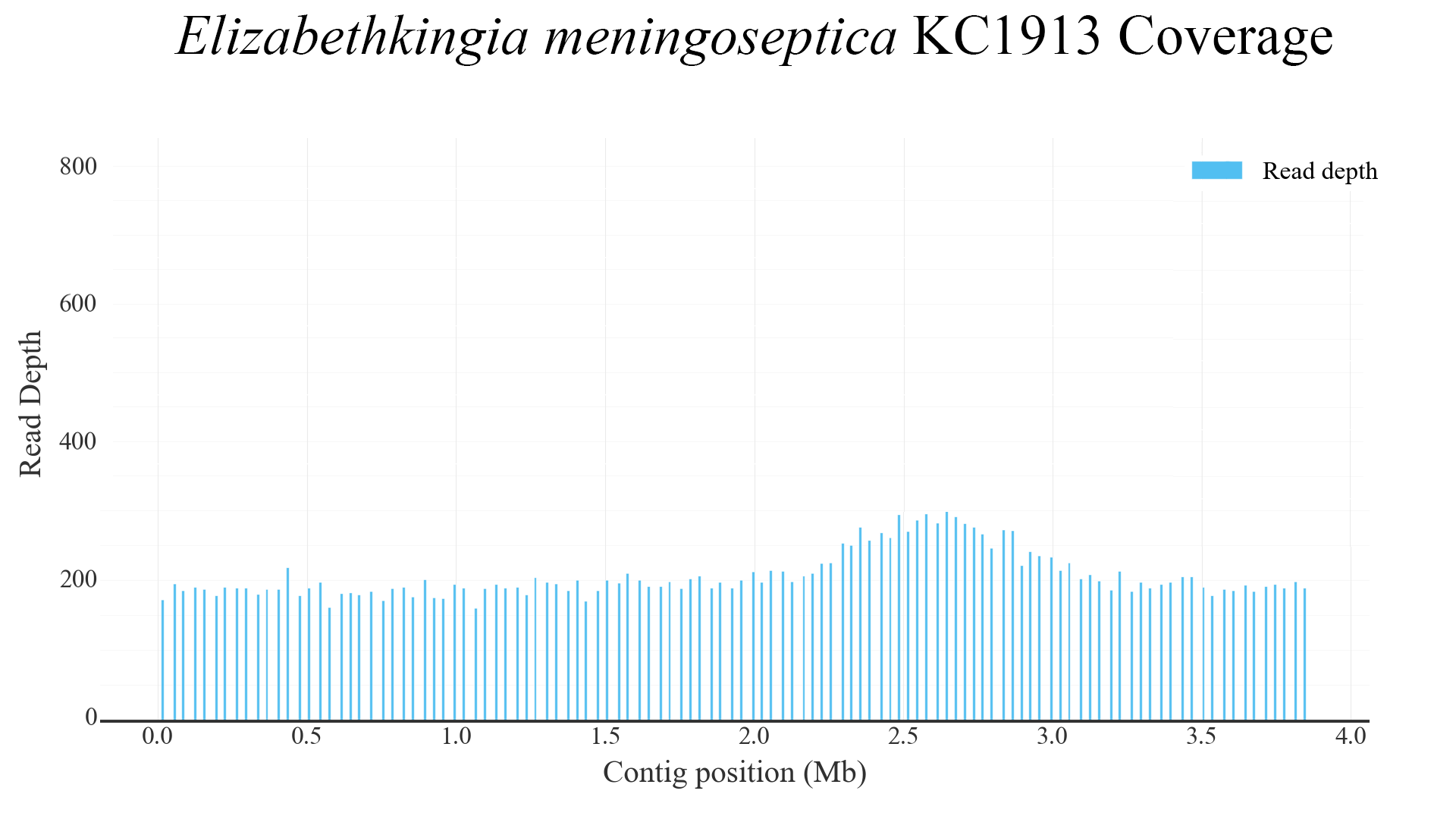

Supplement: FIGURE S2 — The Nanopore R9.4 read coverage for E. meningoseptica KC1913. [file Image_2.tif]
